# Students’ aerobic fitness, self-regulation, and mathematical performance in primary school

**DOI:** 10.3389/fspor.2026.1916893

**Published:** 2026-10-07

**Authors:** Silje Eikanger Kvalø, Megan M. McClelland, Edvin Bru, Sindre M. Dyrstad

**Affiliations:** 1 Department of Education and Sport Science, University of Stavanger, Stavanger, Norway; 2 Human Development and Family Sciences, Oregon State University, Corvallis, OR, United States; 3 Center of Learning Environment, University of Stavanger, Stavanger, Norway

**Keywords:** academic achievement, intervention, off-task behavior, on-task behavior, physical activity

## Abstract

**Purpose:**

This study examined the relationships between aerobic fitness, behavioral self-regulation, and mathematics performance in primary school students, and evaluated the effect of a 10-month school-based Physical activity intervention on self-regulation.

**Methods:**

The Active School study was a ten-month cluster-randomized controlled trial. The sample consisted of 435 students (9–10–years old) in five intervention schools and four control schools. Teachers assessed students’ self-regulation using the Child Behavior Rating Scale (CBRS), at pre- and post-intervention. Aerobic fitness was assessed with a 10-minute interval running test, and mathematical achievement was determined using Norwegian national standardized tests in mathematics.

**Results:**

Partial correlations indicated that aerobic fitness was significantly correlated with self-regulation (*r* = 0.22, *p* < .001) and with mathematics scores (*r* = 0.17, *p* < 0.01). Linear mixed models showed that self-regulation was consistently positively associated with mathematics performance (B = 0.54, (95%) CI [0.44, 0.64], *p* < 0.001). Aerobic fitness was positively associated with mathematics performance in partially adjusted models (B = 0.15, (95%) CI [0.006, 0.23], *p* < 0.001) but this association was no longer significant after adjusting for gender (B = −3.69, (95%) CI [−5.47, −1.19], *p* < 0.001). No significant improvements in students’ behavioral self-regulation compared to the control group were observed.

**Conclusion:**

The findings suggest that while cognitive-behavioral processes strongly drive academic performance, standard school-based physical activity programs may lack the necessary cognitive complexity to enhance independent self-regulatory capacities in fifth-grade students.

## 1 Introduction

Physical activity interventions are associated with multiple physiological, cognitive, and academic benefits to children. However, the magnitude of these effects varies with intervention characteristics (1). Physical Active Learning (PAL) formats, which integrate academic content with movement, have recently been shown to have particular promise for improving academic performance and academic outcomes, especially among students with weaker initial mathematical skills (2) and those with worse health indicators (1, 3). Qualitative and mixed-methods evidence further indicates that PAL can enhance motivation, participation, and classroom behavior, and promote cooperation and social interaction among students (4). Although PAL lessons may progress at a slower pace and cover less curriculum content, both teachers and students report deeper and more sustained learning, highlighting a potential trade-off between breadth and depth of understanding (5). It should be noted that findings from school-based PAL interventions remain mixed, with some studies reporting no main effects on physical activity levels, aerobic fitness, executive function, or academic achievement (2, 6, 7).

A growing body of evidence suggests that embedding physical activity into the school day can improve classroom behavior (8, 9), increase time-on-task (10, 11), and enhance cognitive functioning and academic outcomes (1, 10–13). For example, even brief classroom activity breaks of 4–5 min have been shown to improve on-task behavior and engagement, with cumulative benefits when implemented multiple times across the school day (9, 13). Systematic review evidence further demonstrates that active breaks can positively influence classroom behavior and time-on-task, particularly when delivered at moderate-to-vigorous intensity (14). Physical activity may also benefit attention, particularly among overweight students (15).

A substantial body of research also highlights the importance of aerobic fitness for academic achievement. Higher levels of aerobic fitness are associated with higher grades and improved performance on standardized achievement tests (16, 17), and a meta-analysis demonstrates consistent positive associations between aerobic fitness and academic achievement across reading/language, mathematics, and executive function composite scores (18). The effects of physical activity interventions are shown to be most pronounced for mathematics (1, 19–21). School-based physical activity interventions, particularly those of longer duration and moderate intensity, have also been shown to improve overall academic performance, with stronger effects observed for mathematics outcomes (16). Furthermore, large-scale intervention studies such as the Health Oriented Pedagogical Project (HOPP project) a Norwegian school-based physical activity intervention aimed at promoting public health, reducing cardiometabolic risk factors, and enhancing academic performance, demonstrate that sustained and systematically implemented active learning approaches can produce measurable improvements in academic performance at the population level. These findings, underscore the importance of integrating physical activity as a core component of educational practice rather than viewing it as a short-term initiative (22).

In addition to physical health and fitness, cognitive and behavioral self-regulation skills play critical roles in academic success. Students who can focus, pay attention, and remember instructions are more likely to perform well in school (23). These skills align closely with executive functions, including inhibition, working memory, and cognitive flexibility, which reflect how cognitive processes are enacted in behavior (24). Self-regulation is also associated with long-term outcomes in achievement, interpersonal competence, mental health, and healthy living (25). Within the classroom context, students must navigate specific regulatory demands such as waiting their turn, raising their hand before speaking, and engaging appropriately with academic tasks (26, 27). Higher aerobic fitness may support stronger self-regulation through its association with frontal cortex activation (28). Aerobic fitness also facilitates neurocognitive processes such as attention allocation and semantic processing (29), both of which are critical for mathematical learning (30, 31). Indeed, students with higher aerobic fitness may be more adept at identifying relationships in mathematical problem-solving tasks because of neurocognitive processes involved with the allocation of attentional resources that may underlie scholastic performance (32).

It should be noted that physical activity itself does not affect academic achievement, nor do improvements appear to be driven solely by amount of physical activity (12). Instead, the cognitive demands embedded within physical activity, such as updating rules, inhibiting responses, and shifting attention, appear to be central to gains in self-regulation and executive function (33). Indeed, cognitively engaging forms of physical activity have been shown to exert stronger effects on executive functions than less demanding activities, indicating that aerobic fitness alone is insufficient to drive these changes (1, 34). Combining quantitative (intensity, duration) and qualitative (cognitive engagement) dimensions of physical activity may therefore help students filter distractions and sustain attention (33, 34). Further research is needed to clarify these mechanisms, particularly in older primary school students.

On-task behavior, a classroom-relevant indicator of self-regulation, includes behaviors such as remaining seated, engaging in group work, and responding appropriately to teacher questions (35). PAL interventions have demonstrated positive effects on on-task behavior (4, 8, 35), an important proximal predictor of learning (17). It has been suggested that on-task behavior may diminish over time as intervention novelty subsides (10); however, recent findings indicate that increased engagement during PAL lessons can persist into subsequent lessons, suggesting that the benefits may extend beyond the intervention itself (4). By embedding movement directly into academic content, PAL may help sustain engagement across the school day (10). Physiological responses to exercise may be contributing by increasing levels of hormones such as adrenaline, epinephrine, and norepinephrine, which enhance alertness and readiness to learn (36), while at the same time PAL addresses students’ natural need for movement (37).

Despite a growing body of research demonstrating positive associations between physical activity, aerobic fitness, and behavioral indicators of self-regulation, important gaps remain. While aerobic fitness has been linked to academic performance and cognitive functioning, fewer studies have simultaneously examined its relationship with behavioral indicators of self-regulation in classroom settings. In addition, although interventions such as PAL have shown promise for improving on-task behavior and engagement, there is limited longitudinal and intervention-based evidence examining whether such approaches lead to measurable improvements in self-regulation over time (4, 10). Based on the research summarized above, the present study was guided by a common framework proposing that physical activity and aerobic fitness may support children's self-regulatory and academic functioning. To address this framework, the study first examined the relationships among aerobic fitness, self-regulation, and mathematics achievement and subsequently investigated whether participation in a school-based physical activity intervention could promote improvements in self-regulation over time. The objectives of this study were to:

1. Examine the cross-sectional (baseline) bivariate relationships among self-regulation, aerobic fitness, and mathematical achievement among 5th-grade students in Norway.
2. Examine whether aerobic fitness contributes uniquely to mathematical achievement in a cross-sectional regression model controlling for self-regulation.
3. Investigate whether participating in a 10-month school-based physical activity intervention focusing on physically active learning could improve 5th-grade students’ self-regulation in the classroom over time.

## 2 Method and materials

### 2.1 Participants and study design

Our investigation, called the Active School study, used a cluster randomized controlled trial (RCT) design and included 5th-grade students (9–10 years old) at nine primary schools in Norway. All nine schools were matched based on four criteria (living conditions, activeness in sports, size, and participation in a physically active recess program) and then randomized into two groups (intervention and control group) by the computer program “Researcher Randomizer” (https://www.randomizer.org/). A detailed description of the intervention design, teacher training, and implementation procedures has been reported elsewhere (7, 38), while Figure 1 provides an overview of the weekly intervention dose and component.

**Figure 1:**
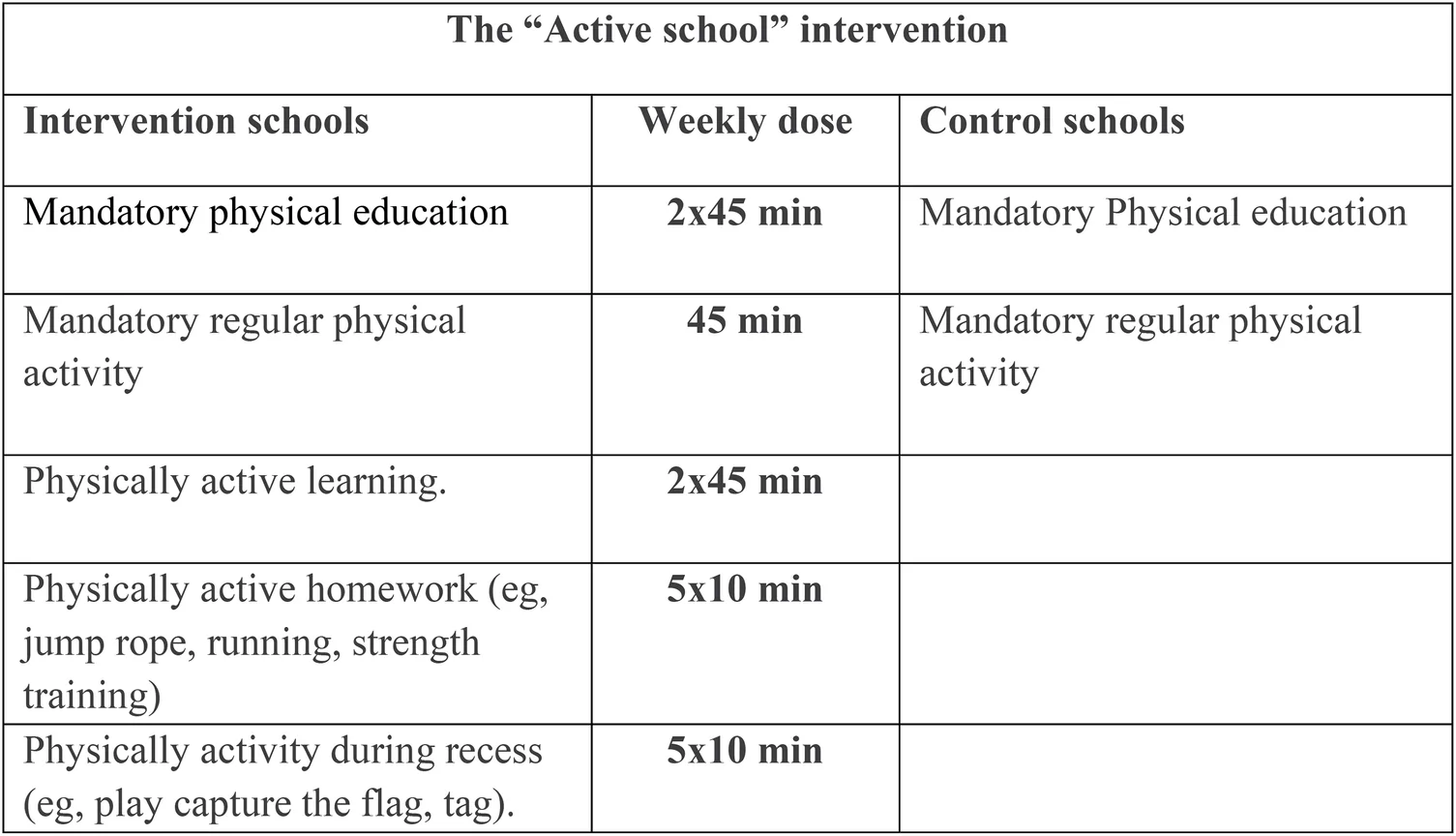
Overview of the intervention model in the active school study.

The intervention was implemented by classroom teachers and consisted of approximately 325 min of physical activity per week, compared with 135 min in control schools. Specifically, it included two weekly 45-minute physically active learning lessons in which movement was integrated into academic subjects, five 10-minute physically active homework sessions, and five 10-minute physically active recess sessions. Examples of activities included movement-based learning tasks, jump rope, running, strength exercises, and organized playground games. The intervention was flexible and adapted to local school contexts while maintaining the prescribed weekly activity dose.

Intervention fidelity data indicated that approximately 89% of the planned intervention activities were implemented. Data were collected in fall (August-September) and spring (May-June) during school days (8 am – 2 pm) for six consecutive weeks. In total, 449 (93%) informed consents were obtained from the students’ parents, and 435 students (223 boys and 212 girls) at 9–10 years old (fifth grade) were included in the testing (see Figure 2). Overall, 16 students dropped out during the intervention period due to sickness, cancellation of participation, or because they moved away from the school district. After accounting for dropouts, the total sample size was 413 students. The study was approved by Norwegian Research Data, now called SIKT (project number 38509).

**Figure 2:**
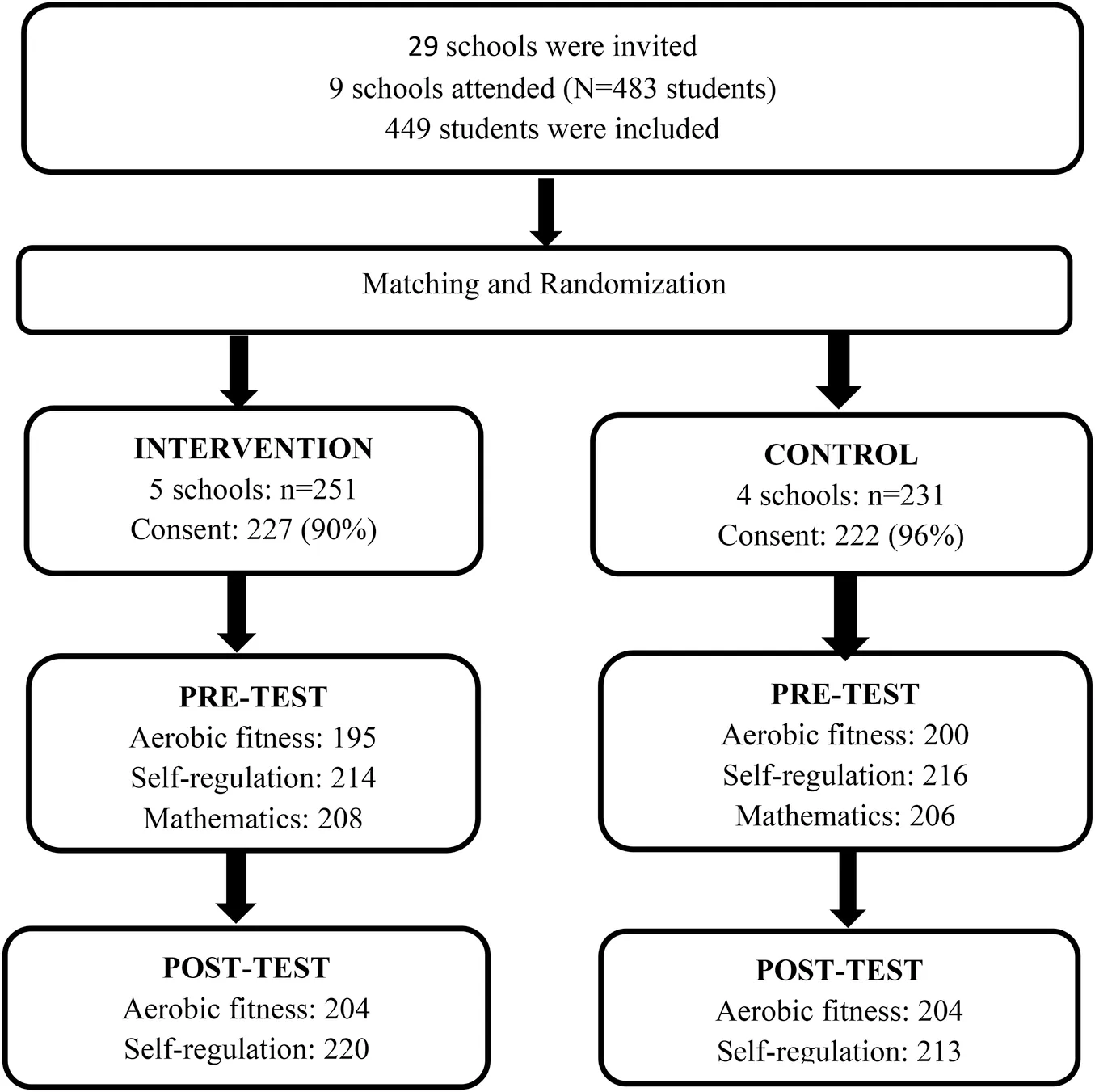
Flow diagram of recruitment and participation of students in the “active school” study.

### 2.2 Self-regulation assessment

The Child Behavior Rating Scale (CBRS) is a teacher-rating instrument that has been demonstrated to be predictive of academic achievement in preschool and kindergarten (39–41), and several studies have documented positive associations between teacher ratings of CBRS and directly measured behavioral self-regulation (27, 41–43). The CBRS is derived from the 32-item Bronson Social Task and Skill Profile, which was designed to help teachers assess students’ goal-oriented classroom behaviors and strategies used to regulate behavior in academic and social situations (40). It has been demonstrated that ten of these items can be used to assess behavioral aspects of self-regulation important for classroom success (39, 42), and these items were therefore chosen for use in the present study.

While the CBRS has primarily been used with younger students, the items are also relevant for older students including those participating in the current study. For example, items such as “Completes learning tasks in an organized way” (inhibitory control), “Observes rules and follows directions without requiring repeated reminders” (working memory), and “Concentrates when working on a task” (attention) are relevant to older students’ classroom behavior because they reflect core self-regulatory skills that remain important throughout the school years.

Each item was rated on a 5-point Likert scale from 1 (never) to 5 (always), with higher scores indicating greater behavioral self-regulation. Consistent with previous work (42), item scores were totaled to create a sum score for each student (scoring ranged from 40 on pre CBRS and 37 on post CBRS). The 10 items were translated and back-translated using standardized procedures and validated by teachers not participating in the present study. To ensure consistency, written explanations were given to the teachers regarding the importance of these measures and instructions for completing the CBRS forms. Teachers completed the 10-item CBRS for each student twice during the intervention period (in autumn and spring).

Several studies have documented positive associations between teacher ratings and directly measured behavioral self-regulation (27, 41–43). In this study the 10-item CBRS demonstrated strong psychometric properties in the current sample. A factor analysis using principal axis factoring and oblique rotation yielded a single factor with factor loadings ranging from 0.82 through 0.91, and a Cronbach's alpha (*α*) = 0. 96. This confirms that the ten CBRS items used assess a uniform construct that likely reflects self-regulation among fifth-grade students as well. Detailed information about the factor analysis is provided in Supplementary materials. We assessed test-retest reliability by calculating the intra-class correlation (ICC) for the variation in CBRS scores across the two time points (44).The ICC for the CBRS was 0.69, indicating moderate to good test-retest-reliability.

### 2.3 Aerobic fitness assessment

Aerobic fitness was assessed using a 10-minute interval running test, with total distance run serving as the outcome measure (45). The test has demonstrated good reliability and validity for assessing aerobic fitness at the group level among 10-year-old students, yielding an intraclass correlation coefficient of 0.84 and explaining 61% of the variance in directly measured VO₂peak, the highest rate of oxygen consumption attained during maximal or near-maximal exercise. This 10-minute interval running test is a reliable and valid tool for determining aerobic fitness on a group level for 10-year-old students, with an overall intraclass correlation coefficient of 0.84, with R^2^ between the estimated VO_2_peak by test and directly measured of 0.61 (46). In this test, the students ran back and forth between two lines on the floor placed 20 meters apart, touching the ground beyond the line with their hands each time they reversed direction. After 15 s, a teacher signaled stop, and the students rested for 15 s before they performed the same task for another 15 s. This process was repeated until a total of 10 min had elapsed. The outcome measurement was total distance run in 10 min. Based on the recommendation by Aadland (47), familiarization tests were performed at each school one week before the main test. The main test was conducted in a gymnasium with a wooden floor, and the students warmed up for 10 min before the test began. Adult test assistants counted the number of laps for each student.

### 2.4 Academic achievement assessment

The Norwegian national tests in mathematics assess the extent to which a school has managed to develop the skills of the students according to the curriculum. The tests are standardized, and all 5th-grade students in Norway take the same tests. In accordance with the national framework of the curriculum, basic skills in mathematics and reading are measured in all subjects. The mathematics test is computer-based and covers three content areas: numbers, measurement, and statistics (48). The Norwegian Ministry of Education and Research administers the test; the national mean score is 50 scale points, standard deviations of 10 are used, and the test is completed in 90 min (48).

### 2.5 Statistical analysis

Preliminary analyses were conducted to ensure no violation of the assumption of normality and linearity.

For the first two research questions, baseline data from the entire sample were analyzed, whereas the third research question involved comparisons between the intervention and control groups. Descriptive statistics and independent samples t-tests were conducted to examine potential gender differences in aerobic fitness and self-regulation. This step was important because previous research has indicated that teachers tend to give girls higher marks than boys on the CBRS (39), which may introduce gender-related bias in self-regulation measures. Consequently, gender was included as a covariate in subsequent analyses to examine whether the observed associations remained after adjustment for potential gender differences. To further examine associations between aerobic fitness, self-regulation, and mathematics achievement, partial correlations controlling for gender were conducted. This approach allowed us to account for potential confounding effects of gender on the observed relationships. Preliminary zero-order correlations indicated small but significant associations between aerobic fitness and self-regulation (*r* = 0.10, *p* = 0.05), and between aerobic fitness and mathematics (*r* = 0.18, *p* < .001). The present correlations suggested that controlling for gender influenced the strength of the association between aerobic fitness and self-regulation, supporting the decision to include gender as a control variable in subsequent analyses.

To investigate the relationship between students’ mathematics skills, self-regulation, and aerobic fitness, a linear mixed model with a hierarchical data structure was used. Mathematics was entered as the dependent variable, whereas self-regulation and aerobic fitness were entered as fixed-effect predictors. Schools were used as random intercepts. The original sample size calculation was based on executive function, the primary outcome of the intervention study. Power calculations indicated that a sample of approximately 250–300 students would provide 80% power to detect a meaningful intervention effect over time. The outcomes examined in the present manuscript were secondary outcomes, and the study was not specifically powered to detect effects on these measures.

Missing data ranged from 2.5% to 11.0% across the study variables: 11 observations (2.5%) for post-CBRS, 14 (3.2%) for pre-CBRS, 18 (4.1%) for mathematics, and 48 (11.0%) for aerobic fitness. Inspection of the data indicated an arbitrary (non-monotone) missingness pattern. To reduce potential bias and preserve statistical power, multiple imputations using a Markov Chain Monte Carlo (MCMC) procedure were performed in IBM SPSS to generate 20 imputed datasets.

Missing data was assumed to be Missing at Random (MAR), as missingness was considered likely to depend on observed participant characteristics and study variables rather than unobserved values. The imputation model included all variables specified in the primary analysis: pre- and post-self-regulation (CBRS) scores, mathematics, aerobic fitness, intervention group, gender, and school affiliation. School affiliation was included in the imputation model to account for the clustered structure of the data during the imputation process.

Finally, a multilevel linear model (linear mixed model) was fitted to each of the 20 imputed datasets, and the parameter estimates were pooled. The model explored whether participating in the Active School intervention affected students’ self-regulation. The post-intervention CBRS score was specified as the outcome variable, with baseline CBRS, intervention group (intervention vs. control), and gender included as fixed effects. To account for the clustered data structure, schools were included as a random intercept, and parameters were estimated using maximum likelihood.

## 3 Results

Descriptive analyses revealed distinct baseline gender differences: boys ran significantly further than girls (mean difference of 55 meters, representing a 5.7% difference), whereas teachers rated girls as having 16.3% higher behavioral self-regulation than boys (Table 1).

**Table 1:** Mean and standard deviation for self-regulation, aerobic fitness, mathematical achievement, and covariates for the entire sample.

Variables	Girls		Boys	
	N	Mean (SD)	N	Mean (SD)
Aerobic fitness (distance - meters)	194	952 (80.04)	202	1007 (105.9)^a^
Self-regulation (CBRS - teacher rating)	207	41.53 (7.41)	223	35.7 (8.74)^a^
Mathematics (scale points)	208	51.58 (9.33)	218	52.36 (10.3)

a Different from girls (<.001).

For the first research question, partial correlations controlling for gender demonstrated that aerobic fitness was significantly associated with self-regulation (*r* = 0.22, *p* < .001) scores and accounted for 4.8% of the variance in teacher-assessed student self-regulation (Table 2). Concurrently, aerobic fitness showed a significant association with scores for mathematics (*r* = .17, *p* = .001) and accounted for 2.8% of the variance in the national test in mathematics. Notably, self-regulation showed a strong association with mathematics, accounting for 24% of the variance in this variable.

**Table 2:** Partial correlation between students’ aerobic fitness, self-regulation, and mathematical achievement for the total sample (*N* = 373).

Variables	Self-regulation	Aerobic fitness
Aerobic fitness (meters)	.22*	
Mathematics	.49*	.17*

* *p* < 0.001. Gender was used as a covariate.

For the second research question, three linear mixed models were conducted for the entire sample with mathematics as the dependent variable (Table 3). Self-regulation was entered into the first model; aerobic fitness was added in the second and gender in the third.

**Table 3:** Linear mixed-effects models predicting mathematics performance from self-regulation and aerobic fitness.

Predictors	Model 1 estimate (*B*) (95% CI)	Model 2 estimate *(B)* (95% CI),	Model 3 estimate *(B)* (95% CI)
Intercept	30.521 (26.16, 34,87)	16.99 (7.79, 26.19)	21.77. (12.38, 31.16)
Self-regulation	0.56 (0,46, 0.66)*	0.54 (0.44, 0.64) *	0.63 (0.52, 0.74) *
Aerobic fitness		0.15 (0.006, 0.23) *	0.008 (−0.001, 0.018)
Gender (covariate)			−3.69 (−5.47, −1.19) *
School variance	73.16	71.30	68.77

*B* = unstandardized regression coefficient (pooled); CI = confidence interval. Model 1 includes self-regulation. Model 2 adds aerobic fitness. Model 3 additionally adjusts for gender. *P* < 0.001*.

The results from Model 1 showed that self-regulation was positive associated with mathematics performance (*B* = 0.56, (95%) CI [0.46, 0.66], *p* < 0.001). In Model 2, self-regulation remained significantly associated with mathematics performance (*B* = 0.54, (95%) CI [0.44, 0.64], *p* < 0.001). Aerobic fitness also demonstrated a positive relationship with mathematics performance (*B* = 0.15, (95%) CI [0.006, 0.23], *p* < 0.001, although its association was weaker than that of self-regulation. In the third model, gender was significantly associated with mathematics performance (*B* = −3.69, (95%) CI [−5.47, −1.19], *p* < 0.001), indicating a systematic difference in baseline scores between boys and girls when holding other variables constant. Across the sequential models, the residual school-level variance decreased from Model 1 (72.61) to Model 3 (68.41), indicating that some of the between-school variance in mathematics performance was accounted for by student-level differences in aerobic fitness and gender.

For the third research question, we examined whether participation in the 10-month PAL intervention was associated with changes in students’ behavioral regulation. A linear mixed-effects model accounting for clustering at the school level indicated that the intervention did not significantly impact the outcome. Controlling for gender, students in the intervention group did not show greater improvements in behavioral self-regulation than students in the control group (*B* = 0.06, 95% CI [−0.40, 0.16], *p* = .25).

## 4 Discussion

This study examined the relationship between aerobic fitness, self-regulation, and mathematics performance in primary school students, as well as the effect of a school-based PAL intervention on self-regulation. Three main findings emerged: (1) aerobic fitness was positively associated with both self-regulation and mathematics performance; (2) aerobic fitness did not uniquely predict mathematics performance when self-regulation was included in the model; and (3) the PAL intervention did not significantly improve students’ self-regulation. Together, these findings contribute to clarifying the mechanisms through which physical activity may influence academic outcomes.

In relation to the first research question, this study demonstrated a significant positive association between aerobic fitness and both self-regulation and mathematics in 5th-grade students. These results support previous evidence suggesting that physical activity and fitness contribute to academic outcomes partly through cognitive and behavioral pathways (1, 17). Although aerobic fitness explained only 4.8% of the variance in self-regulation, even small associations may be meaningful in educational contexts, particularly when accumulated across populations (22). These findings support the notion that physical activity and fitness may contribute indirectly to academic development through cognitive and behavioral pathways (49). Crucially, our finding helps bridge a gap in the literature by linking objective aerobic fitness directly to behavioral indicators of self-regulation within real-world classroom contexts. These results suggest that aerobic fitness is indeed associated with self-regulatory functioning, although the relationship is relatively weak, indicating that fitness alone is unlikely to be a primary driver of self-regulation. For instance, self-regulatory skills fostered by physical activity may contribute more strongly to academic growth over the school year as students encounter new learning opportunities (28). Moreover, students with higher aerobic fitness tend to exhibit stronger executive functions associated with self-regulation, likely reflecting a combination of contextual influences and neurophysiological adaptation (17, 33).

In contrast, the strong association observed between self-regulation and mathematics performance is consistent with prior research identifying self-regulation as a key predictor for mathematics learning in early and middle childhood (25, 26, 50). This finding supports the theoretical argument that core executive functions, such as working memory, inhibition, and attentional control, are fundamental to mathematical learning (50). It also aligns with experimental evidence demonstrating that improvements in self-regulation can drive gains in mathematics performance (42), pointing toward a potentially causal relationship. The positive association between aerobic fitness and mathematics further supports previous findings showing that physical activity and physical fitness are particularly related to mathematics outcomes (18, 19, 21, 51). Together, these results reinforce the idea that physical activity may influence academic performance, but much of this effect is likely mediated through cognitive processes such as self-regulation.

For the second research question, the linear mixed models demonstrated that aerobic fitness did not independently predict mathematics performance once self-regulation was included in the model. This finding directly addresses the relative contributions of aerobic fitness in comparison to cognitively engaging processes. The results suggest that self-regulation is a more proximal and explanatory factor for academic performance than aerobic fitness itself. The substantial magnitude of this relationship, accounting for 22% of the variance, underscores that nearly one-fifth of the individual differences in mathematics performance can be statistically linked to students’ self-regulatory capacities, such as their ability to regulate attention, behavior, and cognitive processes (52). This interpretation is consistent with theoretical accounts emphasizing that cognitive engagement during physical activity, rather than physical activity volume or fitness alone, is critical for improvements in executive function and learning (33, 34) and serve as a central mechanism linking physical activity to academic outcomes (23). In other words, while aerobic fitness may support neurocognitive functioning (29), it is the active application of self-regulatory skills in learning contexts that appears to drive academic performance.

The characteristics of the mathematics test used in this study may further explain the strength of this relationship. Tasks in the test required the integration of working memory, attentional control, and inhibitory processes, and the time constraints may have heightened the importance of efficient self-regulation. Interestingly, the inclusion of gender in the final model revealed that boys performed significantly better in mathematics than girls when holding aerobic fitness and self-regulation constant (B = −3.69, *p* < .05). Given that girls exhibited substantially higher baseline self-regulation (Table 3), controlling for this cognitive advantage unmasks a systematic performance dividend in favor of boys. Factoring in gender and aerobic fitness also reduced the residual school-level variance (from 72.61 to 68.41), suggesting that a portion of the observed differences between schools in mathematics achievement can be attributed to student-level composition regarding gender and aerobic fitness. This highlights the importance of accounting for both demographic characteristics and individual cognitive-behavioral profiles when evaluating academic outcomes within clustered educational environments.

Addressing the third research question, our analyses revealed no significant effect of the 10-month PAL intervention on students’ self-regulation. This lack of an intervention effect mirrors the inconsistencies highlighted in broader literature, where school-based physical activity interventions frequently yield mixed results on executive functions and self-regulation (17). This outcome indicates that merely integrating movement into classroom practice is insufficient for producing measurable improvements in self-regulation. One explanation is that the intervention focused primarily on integrating learning content into movement and emphasized the duration and intensity of physical activity but did not explicitly target self-regulation or include sufficiently cognitively demanding activities. Although the intervention included elements of cognitively engaging and socially interactive activities (e.g., activities that involved decision-making and information processing), these activities may not have provided sufficient cognitive complexity to produce self-regulatory improvements in fifth-grade students (33). This interpretation is consistent with research showing that cognitively engaging forms of physical activity yield stronger effects on executive functions than repetitive and less demanding activities (1, 34). Thus, our findings reinforce the idea that the quality of physical activity, particularly its cognitive demands, is important to include in intervention studies, as well as the quantity. Implementation factors may also explain the lack of intervention effects. As discussed earlier, effective PAL requires systematic integration and adequate teacher support (22). In this study, teachers were responsible for designing and delivering activities, which may have introduced variability in implementation quality (12). Although both teachers and students assert that PAL is a viable pedagogical resource for use in educational settings (5, 38), previous research suggests that it may be challenging for teachers to balance movement with academic content (53). Furthermore, students may initially struggle to manage the simultaneous cognitive and physical demands of PAL tasks (54, 55). Notably, many implemented activities were teacher-directed and repetitive, which may have limited students’ opportunities for autonomy, goal-setting, and strategic thinking. This observation is an important one, given that variation and meaningful engagement are prerequisites for the development of self-regulation, and it is possible that the intervention design may not have fully aligned with its intended pedagogical outcomes. Previous research has cautioned that PAL risks being reduced to mere movement if it is not meaningfully integrated with curricular content (53). The findings presented here might lend support to this concern, and support the need for a more embodied and pedagogically integrated approach to PAL. Moreover, both teachers and students reported that PAL lessons tended to cover less curricular content than traditional instruction, which raises questions about trade-offs between breadth and depth that remain insufficiently examined. Although González-Pérez (5) suggested that PAL includes increased cooperation and social interaction, the educational significance of the PAL intervention depends on how effectively such interactions are structured to support learning goals. Social interaction must be structured in a way that supports educational development, for example, through clear roles and tasks in the groups, and reflection on collaboration as part of the learning process. Finally, the duration of the intervention may have been insufficient for producing measurable changes. While it is well established that PAL improves on-task behavior in the short term (9, 10), evidence for long-term effects on self-regulation is less consistent, and other researchers have found that longer interventions may be required to influence academic and cognitive outcomes (56, 57).

### 4.1 Limitations of the study

Several limitations should be considered when interpreting the findings of this study. First, while teacher ratings provided valuable information but may also have introduced bias. Because teachers were aware of the intervention, their expectations could have influenced their assessments of students’ self-regulation (26). However, if such bias occurred, it would likely have inflated differences between groups, and such differences were not observed. Furthermore, differences in teacher ratings may also reflect classroom-level variation. Including direct assessments of self-regulation in addition to the CBRS would have yielded a more comprehensive evaluation. Nonetheless, significant correlations between CBRS scores and direct measures at pre-test (0.43, *p* < .001) and post-test (0.53, *p* < .001) support the reliability of the assessment procedure and align with previous research (42). Second, ceiling effects on the CBRS may have reduced detectable variability. Although the study sample scored slightly higher on the national mathematics test than the national average (52 vs. 50 points), this difference was modest. Nevertheless, it may indicate that the participating schools performed somewhat above the national average, which could limit the generalizability of the findings to lower-performing school populations. Third, the aerobic fitness test included several rules (for example, touching the ground before turning direction around) that may have been difficult for students with poor self-regulation to follow the test protocol. Lastly, although the cluster-randomized design was statistically accounted for by specifying schools as a random effect in the linear mixed models, the relatively small number of clusters (*n* = 9) schools) may have limited the precision of statistical inference and should be considered when interpreting the findings.

## 5 Conclusion

By addressing both cross-sectional associations and intervention effects, this study contributes to a more comprehensive understanding of how aerobic fitness may support self-regulation and academic development in primary school students. While baseline gender differences were evident, with boys exhibiting higher aerobic fitness and girls demonstrating stronger self-regulation, both aerobic fitness and self-regulation emerged as significant unique predictors of academic achievement in mathematics. Notably, student self-regulation explained a substantial proportion of the variance in national mathematics test scores, underscoring its critical role in classroom success. Conversely, the 10-month PAL intervention did not lead to significant improvements in students’ behavioral self-regulation compared to the control group. These findings suggest that while cross-sectional links between aerobic fitness, cognitive behaviors, and academic outcomes are robust, school-based physical activity interventions may require more targeted or intensive strategies to effectively enhance independent self-regulatory capacities in older children. Future studies should critically examine what constitutes meaningful integration between movement (both quantitative and qualitative) and academic content. More attention should be paid to students’ cognitive and experiential engagement in PAL and to how movement shapes attention, understanding, and self-regulation. In addition, investigating the depth of learning in PAL must be measured as an interaction between cognitive, social, and bodily meaning-making, not just as test scores. Future studies should therefore adopt multi-method approaches that combine conceptually oriented assessments with measures of transfer and long-term retention.

### 5.1 Implications for schools

Taken together, the findings of this study suggest that improving PAL practice requires more than increasing the volume of physical activity within lessons. Rather, it calls for a shift towards pedagogically grounded designs in which movement supports student autonomy, promotes cognitively demanding engagement, and is meaningfully integrated with disciplinary learning goals. Equipping teachers with practical tools and pedagogical strategies for integrating physical activity with learning and self-regulation may enhance the effectiveness of school-based PAL initiatives. Consequently, developing high-quality PAL practice also necessitates more explicit discussion of what is “lost” and what is “gained” in PAL.

## Data Availability

The original contributions presented in the study are included in the article/Supplementary Material, further inquiries can be directed to the corresponding author.
