# Supplementary material for: Students’ aerobic fitness, self-regulation, and mathematical performance in primary school
Source: Front Sports Act Living. 2026 Oct 7;8:1916893. doi: 10.3389/fspor.2026.1916893 (PMC13683658; doi:10.3389/fspor.2026.1916893)
Supplement: Supplementary file 1 [file Supplementaryfile1.docx]

Table: Factor Analysis Results for the Child Behavior Rating Scale (CBRS) Items

| Items from CBRS | Loadings | *h*² | M | SD |
| --- | --- | --- | --- | --- |
| 1 Observes rules and follows directions without requiring repeated reminders. | 0.86 | 0.75 | 3.93 | 0.97 |
| 2 Completes learning tasks involving two or more steps (e.g., cutting and pasting) in organized way. | 0.85 | 0.73 | 4.05 | 0.91 |
| 3 Completes tasks successfully. | 0.87 | 0.76 | 3.95 | 0.94 |
| 4 Attempts new challenging tasks. | 0.84 | 0.70 | 3.83 | 1.04 |
| 5 Concentrates when working on a task; is not easily distracted by surrounding activities. | 0.87 | 0.75 | 3.61 | 1.09 |
| 6 Responds to instructions and then begins an appropriate task without being reminded. | 0.91 | 0.83 | 3.82 | 1.04 |
| 7 Takes time to do his/her best on a task. | 0.81 | 0.67 | 3.91 | 0.95 |
| 8 Finds and organizes materials and works in an appropriate place when activities are initiated. | 0.87 | 0.76 | 4.03 | 0.97 |
| 9 Sees own errors in a task and corrects them. | 0.81 | 0.66 | 3.47 | 1.01 |
| 10 Returns to unfinished tasks after interruption. | 0.89 | 0.79 | 3.72 | 1.03 |
| Eigenvalue | 7.70 |  |  |  |
| Explained variance (%) | 74.4 |  |  |  |
| Cronbachs alpha (α) | 0.96 |  |  |  |

Note. N=448. *h*² = communalities; M = mean; SD = standard deviation
